# Motion artefact reduction in coronary CT angiography images with a deep learning method

**DOI:** 10.1186/s12880-022-00914-2

**Published:** 2022-10-28

**Authors:** Pengling Ren, Yi He, Yi Zhu, Tingting Zhang, Jiaxin Cao, Zhenchang Wang, Zhenghan Yang

**Affiliations:** 1 Department of Radiology, Beijing Friendship Hospital, Capital Medical University, No. 95 Yongan Road, Xicheng District, Beijing, People’s Republic of China; 2 Philips Healthcare, Beijing, China; 3 Shukun (Beijing) Technology Company Ltd., Beijing, People’s Republic of China

**Keywords:** Coronary CT angiography, Motion artefacts, Artificial intelligence, Deep learning

## Abstract

**Background:**

The aim of this study was to investigate the ability of a pixel-to-pixel generative adversarial network (GAN) to remove motion artefacts in coronary CT angiography (CCTA) images.

**Methods:**

Ninety-seven patients who underwent single-cardiac-cycle multiphase CCTA were retrospectively included in the study, and raw CCTA images and SnapShot Freeze (SSF) CCTA images were acquired. The right coronary artery (RCA) was investigated because its motion artefacts are the most prominent among the artefacts of all coronary arteries. The acquired data were divided into a training dataset of 40 patients, a verification dataset of 30 patients and a test dataset of 27 patients. A pixel-to-pixel GAN was trained to generate improved CCTA images from the raw CCTA imaging data using SSF CCTA images as targets. The GAN’s ability to remove motion artefacts was evaluated by the structural similarity (SSIM), Dice similarity coefficient (DSC) and circularity index. Furthermore, the image quality was visually assessed by two radiologists.

**Results:**

The circularity was significantly higher for the GAN-generated images than for the raw images of the RCA (0.82 ± 0.07 vs. 0.74 ± 0.11, *p* < 0.001), and there was no significant difference between the GAN-generated images and SSF images (0.82 ± 0.07 vs. 0.82 ± 0.06, *p* = 0.96). Furthermore, the GAN-generated images achieved the SSIM of 0.87 ± 0.06, significantly better than those of the raw images 0.83 ± 0.08 (*p* < 0.001). The results for the DSC showed that the overlap between the GAN-generated and SSF images was significantly higher than the overlap between the GAN-generated and raw images (0.84 ± 0.08 vs. 0.78 ± 0.11, *p* < 0.001). The motion artefact scores of the GAN-generated CCTA images of the pRCA and mRCA were significantly higher than those of the raw CCTA images (3 [4–3] vs 4 [5–4], *p* = 0.022; 3 [3–2] vs 5[5–4], *p* < 0.001).

**Conclusions:**

A GAN can significantly reduce the motion artefacts in CCTA images of the middle segment of the RCA and has the potential to act as a new method to remove motion artefacts in coronary CCTA images.

## Introduction

**Fig. 1:**
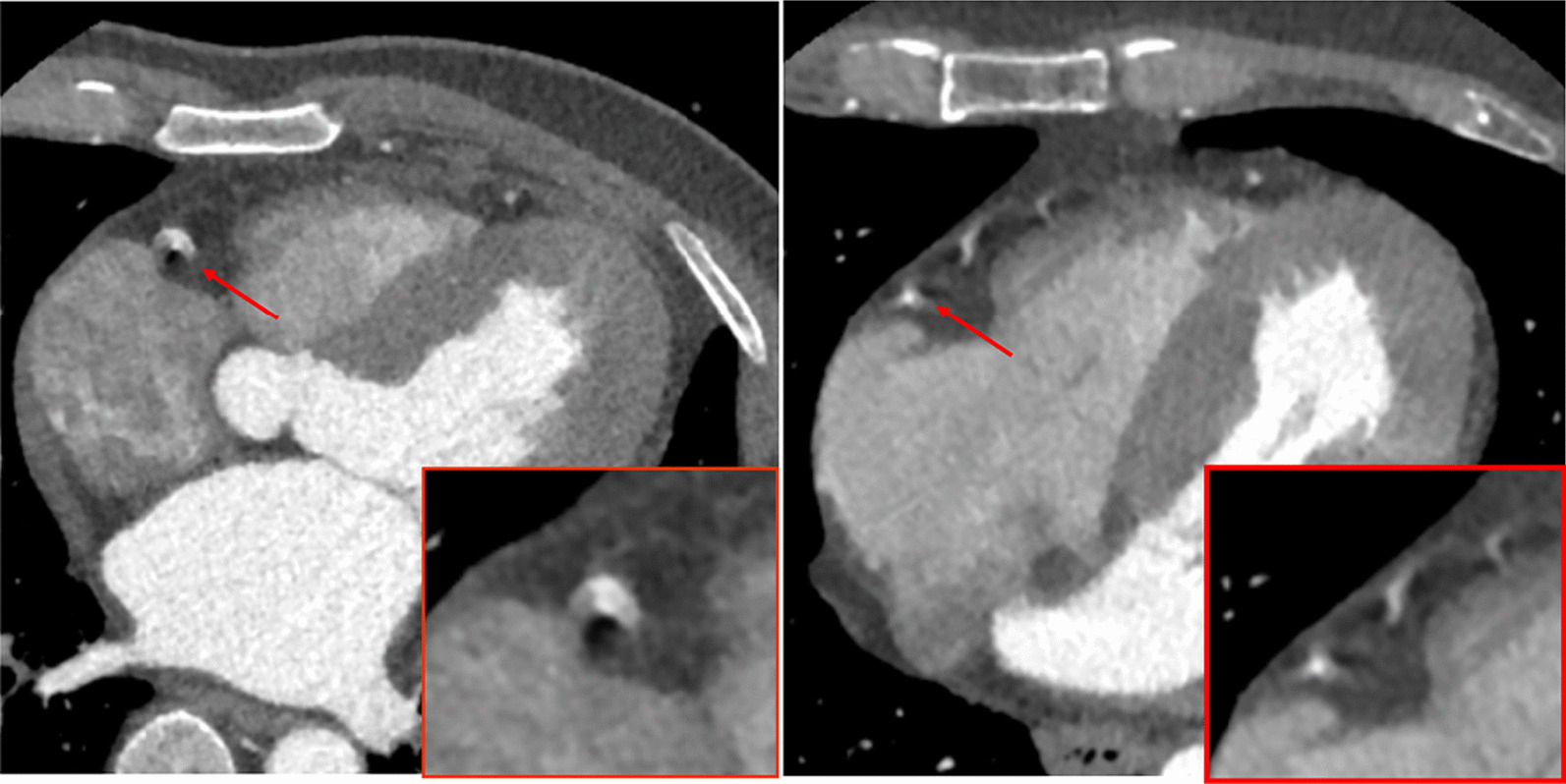
Cardiac motion leads to differently shaped artefacts in CT scans

Cardiovascular disease is now recognized as the leading cause of death and disability worldwide [1]. Coronary computed tomography angiography (CCTA) is widely used for diagnosing cardiovascular disease [2, 3]. Noninvasive CCTA is now poised to become the cornerstone for the evaluation of coronary heart disease and the first diagnostic test in patients with chest pain [4]. However, motion artefacts when the motion speed exceeds the time resolution of the CT equipment degrade image quality and interfere with coronary assessment. Among the branches of the coronary artery, the right coronary artery (RCA) is the most prone to motion artefacts because the direction of motion is perpendicular to the CT scan plane. Although new types of CT equipment with improved hardware capabilities can reduce motion artefacts, high-quality imaging for small and moving vessels is still challenging (see Fig. 1). Motion artefacts potentially limit or even preclude the evaluation of parts of the coronary arteries or cause misinterpretations and are still the main factor affecting the accuracy of coronary CTA diagnosis in clinical practice [5].

Thus, numerous technological solutions have been developed to reduce motion artefacts. Hardware-based solutions, such as dual source CT, have proven useful for improving the diagnostic accuracy at higher heart rates [6]. A motion correction algorithm is another software solution applied to image postprocessing. Previous image processing methods for CCTA motion compensation are based on motion estimation using image registration or the minimization of a motion artefact metric. Methods based on 3D-3D nonrigid image registration have demonstrated excellent motion compensation results [7–9]. However, it is possible that registration is erroneous in the presence of strong motion artefacts, which in turn leads to the degradation of motion compensation. In addition, an iterative motion compensation approach dealing with motion vector field (MVF) estimation that minimizes handcrafted motion artefact measures (MAMs) has been introduced to improve the image quality of coronary arteries [10]. SnapShot Freeze (SSF) is a useful motion correction algorithm that integrates the vessel path and velocity from multiple adjacent cardiac phases to restore the vessel lumen. However, it is a vendor-specific method based on AW4.6 (Advantage Workstations, GE Healthcare) [11, 12].

In recent years, a deep learning method has been applied into the field of medical imaging. Deep learning, in particular, has made it feasible to produce new images using an algorithm known as a generative adversarial network (GAN). A GAN consists of two networks, including a generator and a discriminator, cooperate and compete each other to optimize network parameters [13]. A GAN can generate new synthetic data with much larger diversity which defers from traditional mathematical data augmentation methods. For the purpose of data augmentation, various GAN models have been proposed to generate synthetic images [14–16]. Among them, pix2pix, an image conversion algorithm, learns the relationship between image pairs in order to generate a new image pair based on a single image [16]. Currently, pix2pix can be used for a variety of image-to-image translation purposes; for example, it can convert sketched images to cartoon images or CT images to MRI images [17, 18].

In this paper, a pix2pix network was employed, which generates a motion artefact-free image without depending on image registration or motion compensation. In addition, the image quality of the generated images was evaluated by using subjective and objective methods.

## Materials and methods

### Study participants

This study included retrospectively collected CCTA scans of 97 patients acquired between April 20^th^, 2020, and November 30^th^, 2020. The exclusion criteria were as follows: (1) cardiac surgery, including bypass surgery or percutaneous coronary intervention (PCI); and (2) severe calcification in the CCTA scan. The baseline characteristics of the 97 patients are depicted in Table 1; 55 patients were male, and the median age was 69 years old.

**Table 1:** Characteristics of the patients

Characteristic(*n* = 157)	Value
Age	69(58,72)
Male gender	55(56.70%)
Height(cm)	168.38 ± 8.21
Weight(kg)	74.2 ± 9.12
BMI(kg/m^2^)	25.9 ± 5.86
Heart rate(bpm)	84.94 ± 17.15
Good eating habit (%)	62(63.92%)
Smoker (%)	54(55.67%)
Hypertension (%)	59(62.11%)
Hyperlipaemia (%)	57(58.76%)
Diabetes (%)	45(46.39%)
Family history of coronary heart disease (%)	9(9.28%)
Other past medical history (%)	21(21.65%)

### CTA acquisition

The acquisition of CCTA imaged was performed with a GE Revolution 256-row multidetector CT scanner (GE Healthcare, Waukesha, Wisconsin, US). A prospective electrocardiogram (ECG)-triggered CCTA technique with a 0.625-mm slice thickness was used in the study. Contrast media (iopromide 370 or iohexol 350) was injected into the antecubital vein (60 ml at 5 mL/s for body weights < 100 kg or 80 ml and 6 mL/s for body weights ≥ 100 kg), followed by a 50 ml bolus of saline at 5 mL/s. The raw CCTA images and SSF CCTA images were obtained using AW4.6 (Advantage Workstations, GE Healthcare) after scanning.

### Dataset

In this study, 40 patients were randomly chosen for the training set, 30 patients were randomly chosen for the verification set, and 27 patients were randomly chosen for the test set. For each patient in the datasets, two-dimensional slices, including the proximal RCA (pRCA), mid-RCA (mRCA) and distal RCA (dRCA), were selected from the raw CCTA images and SSF images. Phases with extreme motion artefacts were excluded because segmentation of the coronary artery was impossible.

### Image preprocessing

#### Normalization

The pixel intensities of the CT scans can be expressed in Hounsfield units (HU), which is a standard quantitative scale of radiodensity. All raw data were first converted into HU values. Then, the intensity values of each slice were normalized from [− 300,500] HU to [-1,1]:

$$\mathrm{M}=\frac{HU-MinHU}{MaxHU-MinHU}*2-1$$

where M denotes the rescaled value, HU denotes the original HU value, and MinHU and MaxHU denote the min and max bound values.

#### ROI selection

Before training the GAN network, it is necessary to extract artefact regions of interest (ROIs) using an effective preprocessing method. Based on the unique characteristics of motion artefacts, we calculate the residuals of the raw image and the SSF image in the region of the right crown. Then, we adopt thresholding to obtain the binary image and locate the centroids of the artefacts with a binary morphology method. Finally, all images are cropped with square ROIs to a size of 64 × 64 pixels, and then these ROIs are resized to 256 × 256 pixels.

### GAN framework

In this work, we adopted the pix2pix framework proposed by Phillip et al. [16]. This architecture is an approach for training a generator model and is typically used for generating images. Similar to most GANs, our framework consists of deep convolutional neural network architectures that contain two subnetworks: a single generator network and a single discriminator network. The generator (G) attempts to learn a mapping from the input artefact images to the output artefact-corrected images, and the discriminator (D) learns to discriminate the generated artefact-corrected images and the SSF images (ground truth).

During the training of the GAN, both G and D are learned simultaneously. The discriminator model is trained to classify images as real (from the ground truth dataset) or fake (generated), and the generator is trained to fool the discriminator model. To do this, the following adversarial loss function can be utilized:

1 $${Loss}_{GAN}=arg\underset{G}{\mathrm{min}} \underset{D}{\mathrm{max }} {Loss}_{BCE}\{G(x),y\}+{\lambda Loss}_{L1}\{G\left(x\right),y\}$$

2 $${Loss}_{BCE}={E}_{x,y}\left[\mathrm{log}D\left(x,y\right)\right]+{E}_{x}\left[\mathrm{log}(1-D\left(x,G(x)\right)\right]$$

3 $${Loss}_{L1}={E}_{x,y}[{\Vert y-G(x)\Vert }_{1}]$$

where *x* denotes the artefact images and *y* denotes the SSF images (ground truth). G tries to minimize the adversarial loss, and D tries to maximize it. *λ* is a hyperparameter that balances the contributions of the different loss components.

**Fig. 2:**
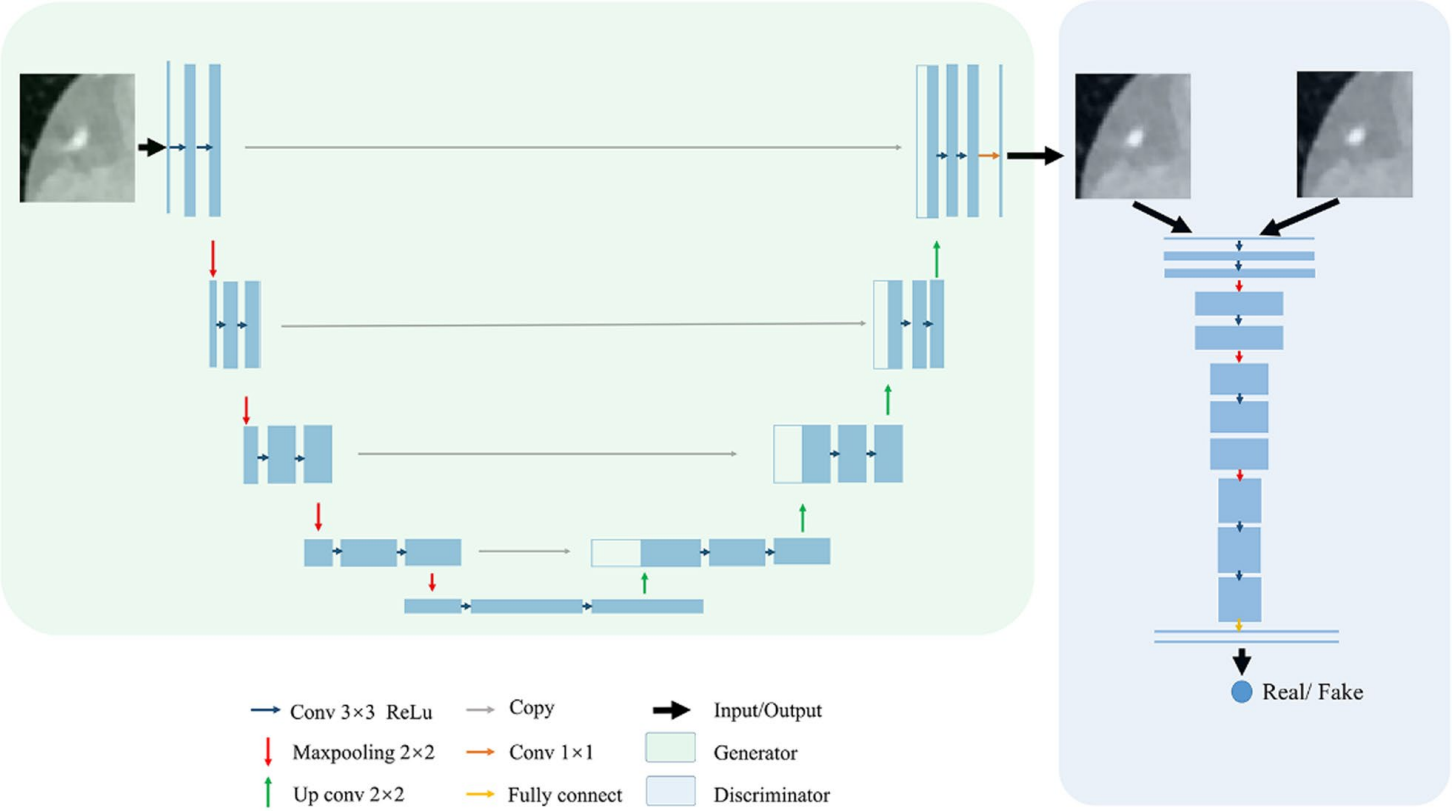
Illustration showing the framework of the GAN model

The specific structure of our method is shown in Fig. 2. The generator is a Res-UNet-based encoder-decoder structure, which combines the advantages of UNet [19] and a deep residual network [20]. UNet adds long skip layers between the downsampling and upsampling layers. This propagation of information from previous layers allows images to retain low-level information and creates sharper results. The convolution block with residual connections can boost information exchange across different layers and can alleviate the vanishing gradient issue. For the discriminator, a modified PatchGAN architecture with VGG16 as the base model is utilized. It tries to classify whether each 70 × 70 pixel patch in an image is real or fake. We run this discriminator convolutionally across the image, averaging all responses to provide the final output of D.

## Experimental setup

All the training and experiments were conducted on a personal computer equipped with an Intel Core i7 7980X CPU with 32 GB main memory and two NVIDIA GTX1080 GPUs. The proposed deep network was implemented using the Keras open-source deep learning library, and TensorFlow was chosen as the backend deep learning engine.

The training procedures lasted for 1000 epochs, and all relevant parameters in the generator and discriminator were simultaneously optimized using the Adam optimizer with a learning rate of 0.0001. The batch size was 4, and *λ* was 10.

### Evaluation of the model

#### Objective image evaluation

To quantitatively compare the quality of raw, SSF and GAN-generated images, three traditional metrics, structural similarity (SSIM), Dice similarity coefficient (DSC) and the circularity, were applied to the binary images representing the segmented vessel region in the three images. SSIM measures the similarity between two images from three aspects: luminance, contrast, and structure [21], which can be written as:

$$SSIM=\frac{\left(2{\mu }_{x}{\mu }_{y}+{c}_{1}\right)\left(2{\sigma }_{xy}+{c}_{2}\right)}{\left({\mu }_{x}^{2}+{\mu }_{y}^{2}+{c}_{1}\right)\left({\sigma }_{x}^{2}+{\sigma }_{y}^{2}+{c}_{2}\right)}$$

where *c*_1_ and *c*_2_ are small constants to stabilize the computation and ${\mu }_{x}$ and ${\sigma }_{x}^{2}$ are the mean and the variance of the images respectively with x and y indicating the different images to compare.

DSC is a commonly used index to evaluate the similarity between two sets of data [22]. The DSC between two binary images can be written as

$$\mathrm{DSC}=\frac{2\sum_{i}^{N} {p}_{i}{q}_{i}}{\sum_{{f}^{i}}^{N} {p}_{i}^{N}{q}_{i}}$$

where *N* denotes the total number of pixels in the image and pi and qi denote the pixel values of the different labelled segmentation samples. For each GAN-generated image and raw CCTA image, the vessel regions are segmented and then compared with the ground truth (SSF images) segmentations using DSC.

On the other hand, a roundness measure was previously proposed to quantify motion artefacts because blood vessels passing through the plane appear circular at rest and deform with motion [23]. The circularity is defined as

$${L}_{circ}=\frac{{p}^{2}}{4\pi A}$$

where *A* and *p* are the area and perimeter of the segmented binary vessel, respectively. The circularity of a perfect circle is equal to one. Since A and p are measured on a pixelized image, the circularity value may be over one in some cases due to discretization errors, especially when the binary vessel area is too small. Therefore, it is necessary to interpolate and enlarge the segmented binary vessel images before calculating the circularity. The ranges of the SSIM values, DSC values and circularity values are 0 ~ 1, and a higher SSIM value, DSC value and circularity value indicate a higher quality.

### Subjective image evaluation

**Fig. 3:**
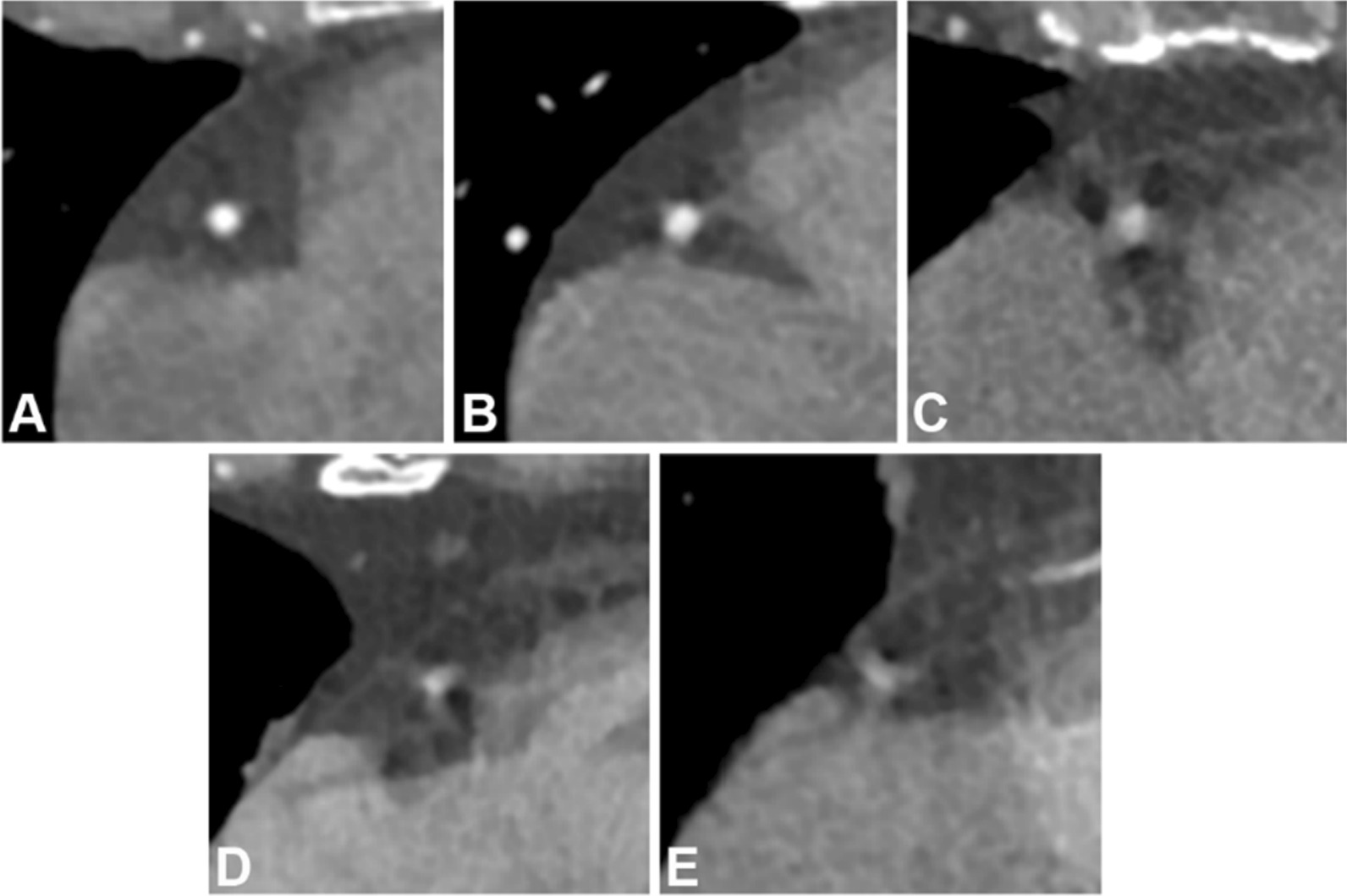
A to E represent the coronary segments scored 5 to 1 in terms of the degree of motion artefacts

Image quality was visually assessed by three observers: two radiologists with 8 and 13 years of experience, respectively. The observers were blinded to the patients' data and the image reconstruction method and assessed the artefact image, freeze image and GAN-generated image in random order. The degree of motion artefacts (1, highly remarkable; 2, remarkable; 3, moderate; 4, minimal; and 5, none, as shown in Fig. 3) and overall image quality (1, nondiagnostic; 2, reduced; 3, adequate; 4, good; and 5, excellent) was rated based on a 5-point Likert scale. The difference was resolved through consensus between these two observers. Interobserver agreement was calculated using intraclass correlation coefficient (ICC). Because the artefacts were difficult to evaluate on separate images, the artefact image, freeze image and GAN-generated image for each patient were simultaneously shown to the radiologists for further evaluation using the overlay function of the FSLeyes viewer (https://fsl.fmrib.ox.ac.uk/fsl/fslwiki/FSLeyes).

## Statistics

The SSIM, DSC, and circularity index and subjective scores were expressed as the mean ± standard deviation (SD) or the median (interquartile range) according to the data normality determined by Kolmogorov–Smirnov test. We used a T test or a Wilcoxon signed ranks test to compare the continuous variables between the artefact images and GAN-generated images. ANOVA test or Kruskal–Wallis H test was used to compare the continuous variables among the three groups. Statistics were computed using R (version 3.2.1; http://www.r-project.org/). The significance was set to P < 0.05 (2-sided).

## Results

### Objective image quality

**Fig. 4:**
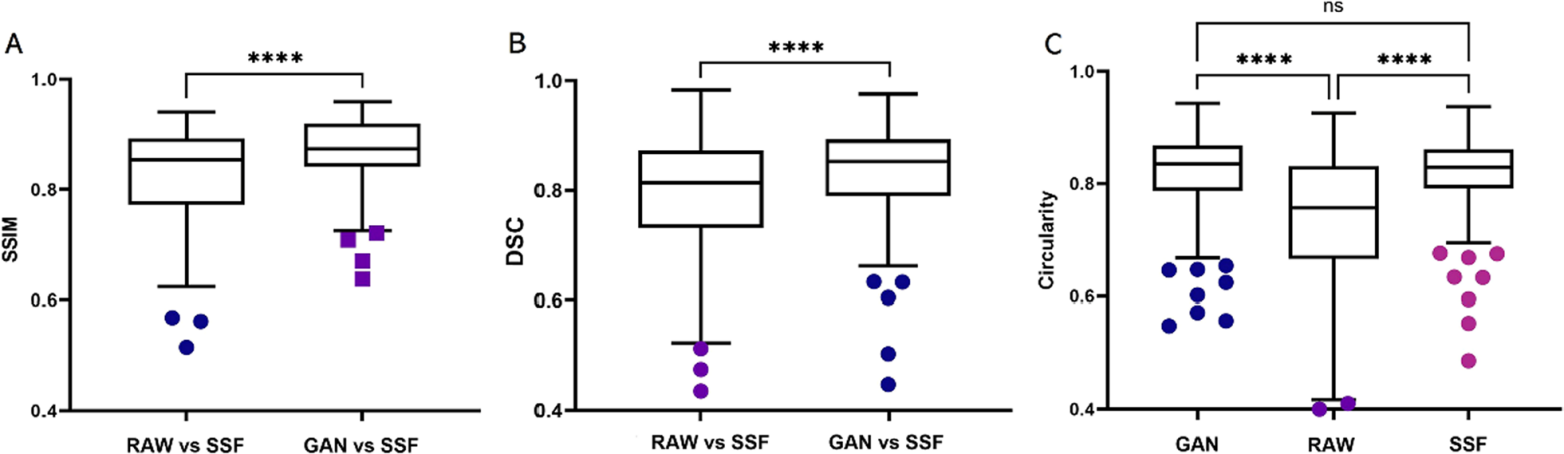
The structural similarity (**A**), Dice similarity coefficient (**B**) and circularity index (**C**) of the raw, SSF and GAN-generated CCTA images (SSIM: structural similarity, DSC: Dice similarity coefficient)

**Fig. 5:**
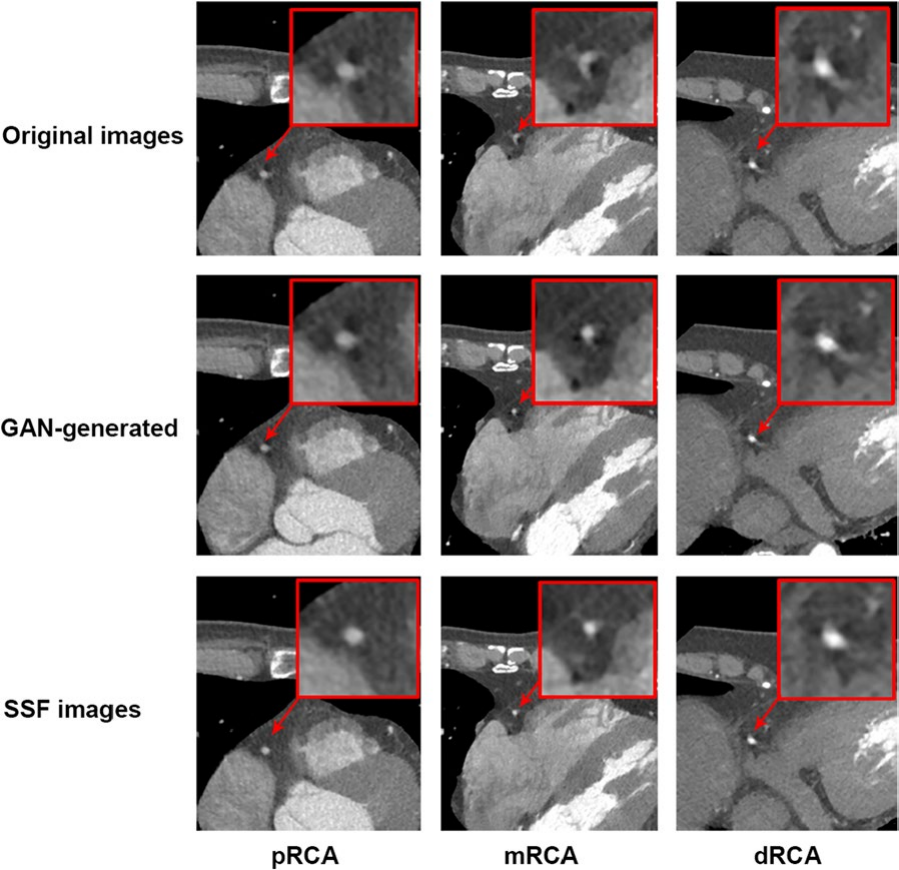
The representative images of the pRCA, mRCA and dRCA in Raw, GAN-generated, and SSF CCTA images

Figure 4 shows the quantitative analysis results. Among them, the normalized circularity was significantly higher for the GAN-generated images than for the raw motion-affected images of the RCA (0.82 ± 0.07 vs. 0.74 ± 0.11, *p* < 0.001), and there was no significant difference between the GAN-generated images and SSF images (0.82 ± 0.07 vs. 0.82 ± 0.06, *p* = 0.96). Moreover, the results for the DSC showed that the overlap between the GAN-generated and SSF images was significantly higher than the overlap between the GAN-generated and raw motion-affected images (0.84 ± 0.08 vs. 0.78 ± 0.11, *p* < 0.001). Furthermore, the GAN-generated images achieved the SSIM of 0.87 ± 0.06, significantly better than that of the raw motion-affected images 0.83 ± 0.08 (*p* < 0.001). Figure 5 shows some representative patient images. The first line shows the ROIs of the raw images with motion artefacts. The second line shows the ROIs of the GAN-generated images after motion artefact removal. The third line shows the ROIs of the SSF (ground truth) images.

### Subjective image quality

In the test set of CCTA images, ICC was 0.89 (95% confidence interval: 0.85–0.91), which indicated excellent interobserver agreement. Moreover, the motion artefacts reduction scores of the GAN-generated CCTA images of the pRCA and mRCA were significantly higher than that of the raw CCTA images (3 [4-3] vs 4 [5-4], *p* = 0.022; 3 [3-2] vs 5[5–4], *p* < 0.001) (Table 2). No significant difference in motion artefacts reduction score was identified in dRCA between the raw and GAN-generated images. Furthermore, the overall image quality score of the GAN-generated CCTA images of the pRCA, mRCA and dRCA were significantly higher than that of the raw CCTA images (Table 2). During the simultaneous evaluation of the raw and GAN-generated CCTA images, the radiologist agreed that although artefacts were still visible in some parts of the RCA in the GAN-generated CCTA images, the RCA looked grossly swollen in all the raw CCTA images when compared with the GAN-generated CCTA images.

**Table 2:** Subjective image evaluation between of the raw and GAN-generated CCTA images in the test set of coronary CT angiography

Segment		Raw	GAN- generated	*p* value
	Motion artefact score, median (interquartile range)	3 (4–3)	4 (5–4)	0.022
pRCA	1, highly remarkable, *n* (%)	0	0	
	2, remarkable, *n* (%)	2/25(8%)	0	
	3, moderate, *n* (%)	13/25(52%)	7/25(28%)	
	4, minimal, *n* (%)	10/25(40%)	12/25(48%)	
	5, none, *n* (%)	0	6/25(24%)	
	median (interquartile range)	3 (3–2)	5(5–4)	*p* < 0.001
mRCA	1, highly remarkable, *n* (%)	3/25(12%)	1/25(4%)	
	2, remarkable, *n* (%)	3/25(12%)	0	
	3, moderate, *n* (%)	14/25(56%)	1/25(4%)	
	4, minimal, *n* (%)	5/25(20%)	10/25(40%)	
	5, none, *n* (%)	0	13/25(52%)	
	median (interquartile range)	4 (4–3)	4 (4–4)	0.822
dRCA	1, highly remarkable, *n* (%)	1/25(4%)	0	
	2, remarkable, *n* (%)	2/25(8%)	1/25(4%)	
	3, moderate, *n* (%)	6/25(24%)	4/25(16%)	
	4, minimal, *n* (%)	14/25(56%)	17/25(68%)	
	5, none, *n* (%)	2/25(8%)	3/25(12%)	
	Overall image quality score, median (interquartile range)	3 (4–3)	4 (5–4)	*P* < 0.001
pRCA	1, nondiagnostic, *n* (%)	0	0	
	2, reduced, *n* (%)	1/25(4%)	0	
	3, adequate, *n* (%)	13/25(52%)	6/25(24%)	
	4, good, *n* (%)	11/25(44%)	12/25(48%)	
	5, excellent, *n* (%)	0	7/25(28%)	
	median (interquartile range)	3 (3–2)	4 (4–3)	*P* < 0.001
mRCA	1, nondiagnostic, *n* (%)	2/25(8%)	0	
	2, reduced, *n* (%)	6/25(24%)	2/25(8%)	
	3, adequate, *n* (%)	14/25(56%)	10/25(40%)	
	4, good, *n* (%)	3/25(12%)	11/25(44%)	
	5, excellent, *n* (%)	0	2/25(8%)	
	median (interquartile range)	4 (5–3)	5 (5–4)	*P* < 0.001
dRCA	1, nondiagnostic, *n* (%)	0	0	
	2, reduced, *n* (%)	3/25(12%)	1/25(%)	
	3, adequate, *n* (%)	4/25(16%)	4/25(16%)	
	4, good, *n* (%)	11/25(44%)	7/25(28%)	
	5, excellent, *n* (%)	7/25(28%)	13/25(52%)	

## Discussion

To our knowledge, our study is the first to use a pix2pix-based algorithm to improve CCTA image quality, which is a novel solution for CCTA motion correction. Here, the trained pix2pix GAN successfully corrected CCTA images with motion artefacts.

Because of the complexity of motion artefacts, it is difficult to directly quantify the image quality of coronary artery motion artefacts, so we referred to the previous methods and tried to quantify the severity of motion artefacts using subjective and indirect quantitative index evaluations.

The subjective evaluation was performed by two experienced radiologists at the RCA location. This evaluation index may be biased due to the subjective factors of the radiologists. Nevertheless, it is still the most convincing evaluation of coronary arteries. For the evaluation scores, the radiologists agreed that the GAN-generated image scores were significantly improved, artefact suppression could be observed in the pRCA and mRCA images. Moreover, all corrected images quantitatively demonstrated improved quality, with SSIM, Dice and circularity being significantly higher in the RCA of the GAN-generated images than those of the raw CCTA images; that is, the GAN-generated images were more accurately replicated the true CCTA images. Furthermore, there was no significant difference between the corrected images and the reference images, meaning that the GAN-generated images were sufficiently similar to the SSF images.

Although current techniques have been successful for motion correction, they are generally appropriate only for specific applications. For example, previous studies have proven that SSF is a promising method for eliminating motion artefacts and improving image quality. However, this is a vendor-specific method, which means it may not be applied to other CT systems from different vendors. Moreover, compared with the traditional iteration method, the deep learning method is simpler in operation and faster in processing time.

A deep learning approach for motion correction may be more generally applicable, as it is entirely a post-processing method and does not require any motion measurements during scanning. Specifically, there are several advantages to our method. Firstly, a residual U-Net framework can learn features that are added to or subtracted from the input image instead of learning the entire output image, which not only makes deeper networks easier to train, but also allows learning more details and information. Secondly, for that the coronary artery occupies only a very small region of the whole CCTA image, it is difficult for the GAN model to find the location of the motion artefact. Thus, an automatically method was designed to locate the motion artefacts and cut the ROI. What's more, the results of preliminary experiment showed that the texture and edge information of the heart region in the input images is necessary for the GAN model, and the ROI size of 64 × 64 pixels is the most suitable size. Thirdly, compared with a traditional L1 loss function, the discriminator is a “variational” loss function with adjustable parameters, and the results show better spatial consistency, as well as did not generate additional motion artefacts. This property is essential for medical imaging because it will not mislead clinicians. Finally, actual learning was performed on 2D patches of the coronary artery, which means that a large amount of data can be obtained.

The present study still has some limitations. Firstly, data from only 1 scanner were used in this study. Hence, future studies should assess the generalizability of our results using more patients and several scanners. Secondly, only 40 cases were used for training the deep learning algorithm in this study. Considering the large number of slices per case, we thought that our datasets could substantially cover the possible signal distributions of the raw CCTA data. However, this assumption should be confirmed by checking the signal distributions of large datasets. In the future, the variants of the deep learning algorithm in our study should also be applied and validated on diseases with various appearances. Thirdly, the CCTA images with heavily calcified segments which were featured with major movement artefacts were not included in this study. This type of images was typical for a clinical radiologist when dealing with movement artefacts and should be included in the future study to enrich the diversity of the dataset. Finally, our method performs worse at the dRCA than at the pRCA and mRCA because coronary motion artefacts can be classified into different patterns of vessel deformation in different locations. Therefore, in the future, we should design different deep learning models for different artery parts.

In conclusion, we successfully improved the artefact CCTA image quality using a GAN model and created GAN-generated images that have contrast similar to that of SSF images with fewer motion artefacts in the RCA while preserving lesion contrast. The proposed GAN-based algorithm may facilitate the introduction of synthetic CCTA imaging into clinical practice.

## Data Availability

The data of this study are available from the corresponding author upon reasonable request.

## Abbreviations

CCTA: Coronary CT angiography
pRCA: Proximal right coronary artery
mRCA: Mid-right coronary artery
dRCA: Distal right coronary artery
GAN: Generative adversarial network
SSIM: Structural similarity
DSC: Dice similarity coefficient
SSF: Snap shot freeze
